# Modeling and Analysis of Radial Electromagnetic Force and Vibration Characteristics Based on Deflection Dual-Stator Switched Reluctance Generator

**DOI:** 10.3390/mi13091494

**Published:** 2022-09-08

**Authors:** Zheng Li, Libo Liu, Pengju Wang, Yu Liu, Xiaopeng Wei, Qianqian Xu, Hexu Sun

**Affiliations:** School of Electrical Engineering, Hebei University of Science and Technology, 26 Yuxiang Street, Shijiazhuang 050018, China

**Keywords:** switched reluctance generator, finite element analysis, electromagnetic radial force, inherent frequency, electromagnetic vibration

## Abstract

In this paper, a mechanical model of the deflection dual-stator switched reluctance generator (DDSRG) is developed, and the advantages of the dual-stator structure for the deflecting motion are analyzed. Secondly, the spatio-temporal and spatial distribution characteristics of the inhomogeneous electromagnetic force are derived analytically and further verified by fast Fourier transform (FFT).Thirdly, the spatial and temporal distributions of electromagnetic forces of DDSRG are calculated based on finite element software, and the distributions of electromagnetic forces under different motion states are analyzed. By combining the analysis of modal analysis and harmonic response analysis, the free mode and vibration response acceleration variation laws of the internal and external stator are determined. The results show that the order of electromagnetic forces on the stator at rated speed is mainly 8 times the fundamental frequency, and the modal vibration order is more violent in the order of 2–7. Finally, the experimental platform of DDSRG is built, and the vibration characteristics are tested to verify the validity and accuracy of the proposed simulation results.

## 1. Introduction

Energy problems and environmental issues in the 21st century have become one of the world’s major concerns, and switched reluctance motors (SRMs) have received widespread attention from scholars around the world by virtue of their simple structure, high fault tolerance, wide speed range, and reliable operation [1,2]. With the development of modern power electronics and vehicle drive technology, SRMs are particularly suitable where the reliability of drive systems is required, such as in electric vehicles, aerospace power systems, and wind power generation [3,4,5]. However, the special structure of SRMs with biconvex poles and the nonlinear current excitation method cause large vibration and noise, so the vibration constraint of SRMs is more demanding, which makes the widespread use of SRMs a great challenge [6,7,8].

The world’s methods for solving SRM radial force analysis and vibration problems are roughly divided into two types: drive control changes and motor mechanical structure optimization [9,10,11]. From the perspective of motor drive control analysis, some researchers have proposed a two-pole commutation active vibration-cancellation control using negative voltage pulse width modulation (PWM).This method can not only effectively reduce the vibration occurring on the stator during commutation but also keep the torque ripple and copper loss of the motor within a reasonable range [12,13].The optimization of the voltage fluctuations on the PWM control method does not trigger natural frequency vibrations and has a better damping effect at low- and medium-speed conditions [14,15]. In recent years, the literature [16,17] has proposed the introduction of direct force control and reference current adapters based on torque control, and this control strategy is effective in reducing vibrations under steady and transient conditions. This method slightly modifies the torque ripple-minimizing reference current, and torque fluctuations remain almost constant, but the accuracy of radial force determination is a major obstacle to the implementation of this technique [18].

To reduce the vibration and noise of SRMs, prediction of vibration and noise is indispensable in the process of motor design [19,20]. Since the SRM works differently from other motors, its electromagnetic force variation pattern with time is closely related to the body structure. Although the direct measurement of SRM vibration characteristics is simple, the disadvantages are the low accuracy of the results and the high cost of construction [21,22]. In recent years, the establishment of simple and accurate analytical models of electromagnetic forces has become a hot research topic, and the calculation of electromagnetic forces by analytical methods can provide help for vibration prediction. To improve the accuracy of computational simulation, the literature [23] proposed a new analytical method of simulation through 3D data in this paper, i.e., considering the excitation of high axial order vibration modes and simulating the vibration law in the 3D domain. Considering that the analysis of the SRM vibration process involves a variety of physical fields, the electromagnetic forces of the stator teeth are calculated based on finite element analysis in the three-dimensional electromagnetic field, and the modal analysis and harmonic response analysis are performed through the laws in the frequency domain to estimate the intrinsic frequency and vibration of the whole motor [24]. In the literature [25,26], the analysis of the SRM multiphysics field and the mutual coupling SRM were carried out for comparison, and a good prediction of the vibration model was calculated by the mode superposition method considering electromagnetic characteristics, mechanical vibrations, and acoustic noise. However, the inhomogeneous electromagnetic forces on the stator teeth and the orthogonal anisotropy of the motor material structure can introduce large errors in the prediction of the vibration and motor modes.

**Figure 1:**
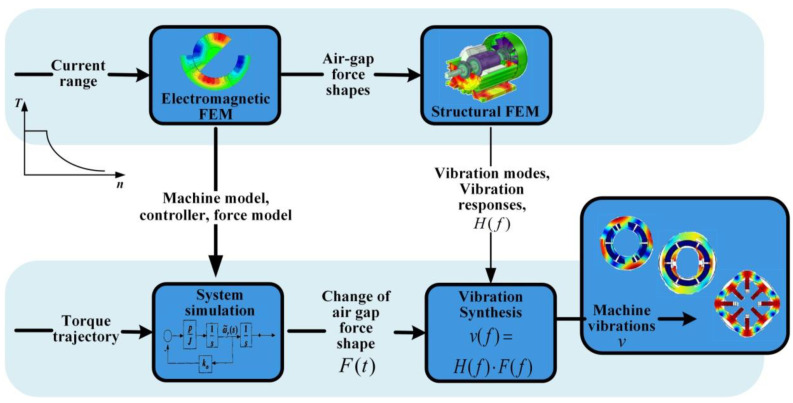
Electromagnetic-vibration analysis framework diagram.

The DDSRG studied in this paper is a generator with a deflectable structure and higher power-generation efficiency [27,28]. In the Section 1, a mechanical model of the DDSRG is developed to illustrate the advantages of the dual-stator structure compared to the common motor structure. In Section 2, a mathematical model of the electromagnetic field of the generator is developed, and the time and space distribution characteristics of the inhomogeneous electromagnetic force are analytically derived and decomposed for verification. In the Section 3, the DDSRG electromagnetic model is analyzed based on finite element software, and the electromagnetic and mechanical characteristics of the generator’s internal and external circuits are analyzed in detail. Then the vibration characteristics are predicted by comparing the analysis of the modal characteristics of the motor stator with the vibration process under electromagnetic force loading. Figure 1 shows the framework of electromagnetic-vibration analysis in this paper. The overall electromagnetic-vibration characteristics of the motor are a combination of the electromagnetic field and modal analysis, and the finite element method is used to analyze the electromagnetic force distribution and the stator–rotor vibration distribution. The overall analysis process includes electromagnetic force finite element analysis, structural transfer function construction, and stator–rotor vibration coupling analysis. The advantage of this analysis method is that it improves the accuracy of the electromagnetic vibration analysis while ensuring the accuracy of the model mesh and parameters, and it is also applicable to the vibration evaluation of other motors. Finally, the experimental platform of DDSRG is built, and the vibration characteristics are tested to verify the validity and accuracy of the proposed simulation results.

## 2. Design and Principle of DDSRG

**Figure 2:**
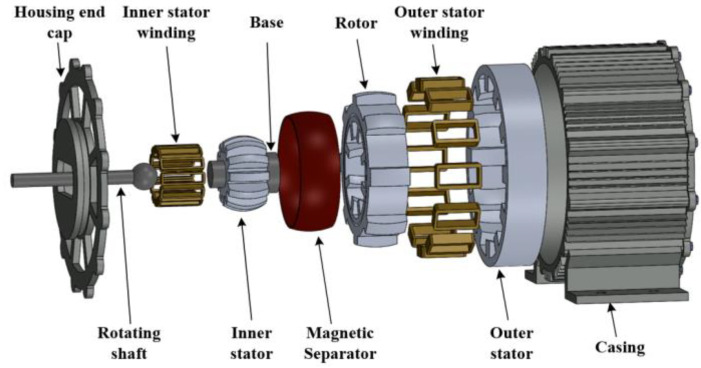
Overall structure distribution of DDSRG.

As a new type of wind turbine, the switched reluctance generator has the advantages of simple structure, low starting wind speed, high power-generation efficiency, easy rectifier storage, and good fault tolerance. Due to the special double-stator structure of the DDSRG, which combines the compactness and fast system response of conventional generators with alternating inner and outer stator operation, the generator power density and power-generation efficiency can be increased. The DDSRG has the advantage of multi-degree-of-freedom operation at constant-rated generator speed, making full use of wind energy for power generation, increasing efficiency and reducing power-generation costs. The specific mechanical parameters of DDSRG are shown in Table 1. Figure 2 is the overall structure distribution map of DDSRG, which is composed of motor shell, stator and rotor, and shaft and base. The advantage of DDSRG is that it has an internal and external double-stator structure and can realize the deflection operation of the motor through the double-stator structure. When the wind direction changes, it can still maintain a good power-generation efficiency. Because the dual stator of DDSRG is composed of internal and external independent loops, magnetic field interference of the internal and external parts of the rotor is prevented by adding an excellent magnetic insulation plate in the middle of the rotor. The bearing connects the rotor through the shaft to realize the motor deflection, reduce the mechanical loss caused by the friction of the motor, and improve the motor efficiency. The shape of internal and external stator teeth is concave spherical, and the shape of rotor tooth pole is convex spherical. It is thanks to the spherical structure design that DDSRG can perform deflection motion.

**Table 1:** Mechanical parameters of DDSRG.

Parameter	Value
Rated speed	200 r/min
Rated power	2.5 kW
Rated torque	70 N_*_m
Rated voltage	380 V
External diameter of external stator	300 mm
Inner diameter of inner stator	13.5 mm
Rotor outer diameter	108 mm
Rotor inner diameter	43.3 mm
Air-gap interval	2 mm
Rotor pole	8
Stator pole	12
Rotor deflection angle	0~17°
Thickness of magnetic insulation board	15 mm

**Figure 3:**
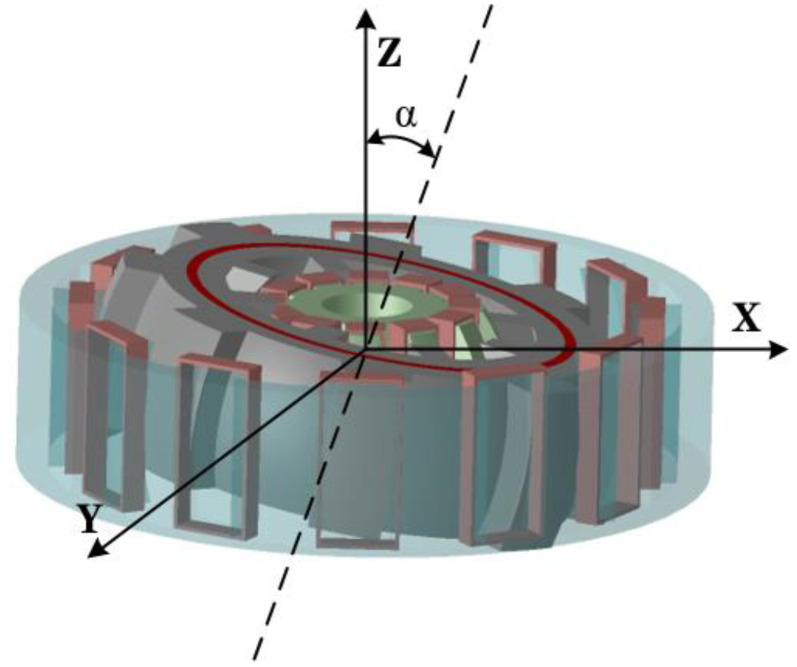
DDSRG deflection state diagram.

**Figure 4:**
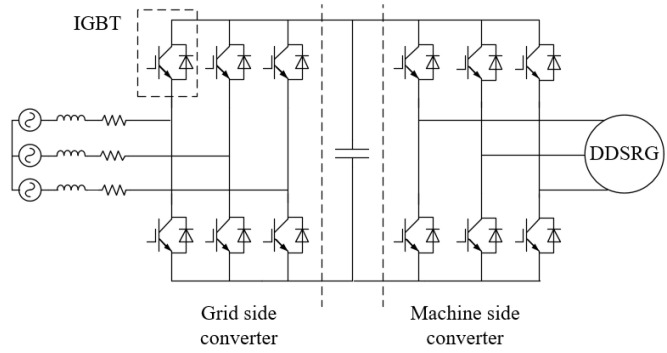
DDSRG power converter.

Figure 3 shows the DDSRG deflection diagram. According to the characteristics of the double stators of the motor, the deflection angle α is the angle between the central shaft of the rotor and the central shaft of the whole generator, and the rotor can be deflected in a certain space. The windings of DDSRG internal and external stator and rotor systems are connected to an independent external circuit, respectively, and the motor efficiency is improved by controlling the deflection state of the motor. The power converter is shown in Figure 4. The rectifier and inverter can be realized by using the dual PWM converter. The generator side is the grid side converter, and the machine side converter is near the generator side. If the converter on one side is disturbed, it will not affect the normal operation of the other side and can better adapt to the fault of the power grid.

## 3. Analytical Calculation of Radial Electromagnetic Force and Magnetic Field

The doubly salient structure of SRM and the switching characteristics of winding current lead to the obvious nonlinear characteristics of electromagnetic force during operation. The vibration of DDSRG in the deflection state is more complex, and the research on the analytical model of electromagnetic torque and electromagnetic force of motor is the premise to solve the vibration problem of stator and rotor. Since DDSRG has two independent loops for driving and deflection, the magnetic circuit is asymmetric, so the process of magnetic circuit analysis will be more difficult. Different from the vibration process analysis of ordinary single-degree-of-freedom motor, the vibration analysis of DDSRG needs to be extended to the calculation of spatial electromagnetic force distribution so as to accurately obtain the vibration characteristics of the motor under different operating conditions. Therefore, this paper calculates and deduces the vibration of DDSRG in time and space.

**Figure 5:**
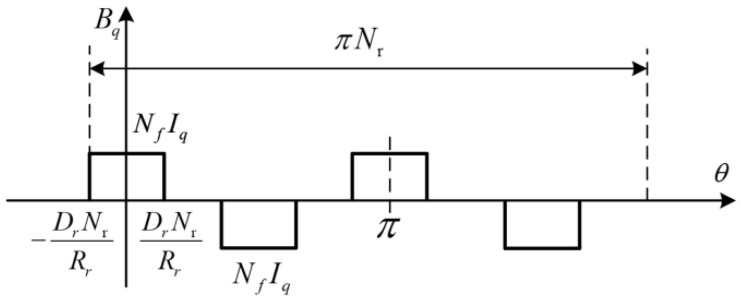
Spatial distribution of rotor magnetomotive force (electric angle).

Because the power-generation characteristics of internal and external circuits are similar, this paper takes the motor external stator and rotor circuit as an example. Assuming that the permeability of the stator rotor magnet is not saturated, Figure 5 is the *q*-term magnetomotive force distribution map of DDSRG, *N_r_* is the number of rotor poles of the motor, *R_r_* is the outer diameter of the outer rotor, *D_r_* is the width of the single-tooth pole of the outer rotor, and *N_f_* is the number of turns of the stator single-tooth pole winding.

The *q*-phase permeability of DDSRG outer rotor can be expressed as the product of winding magnetomotive force and winding current. The magnetomotive force *B_q_* of the *q*-phase tooth pole can be expressed as [29]:

(1) $$B_{q}(\theta )=\left\{ \begin{array}{cc} N_{f}I_{q}(t)- & \;\;\;\frac{D_{r}N_{r}}{R_{r}}\leq \theta <\frac{D_{r}N_{r}}{R_{r}}\\ -N_{f}I_{q}(t) & \;\;\;\frac{\pi N_{r}}{4}-\frac{D_{r}N_{r}}{R_{r}}\leq \theta <\frac{\pi N_{r}}{4}+\frac{D_{r}N_{r}}{R_{r}}\\ N_{f}I_{q}(t) & \;\;\;\frac{\pi N_{r}}{2}-\frac{D_{r}N_{r}}{R_{r}}\leq \theta <\frac{\pi N_{r}}{2}+\frac{D_{r}N_{r}}{R_{r}}\\ -N_{f}I_{q}(t) & \;\;\;\frac{3\pi N_{r}}{4}-\frac{D_{r}N_{r}}{R_{r}}\leq \theta <\frac{3\pi N_{r}}{4}-\frac{D_{r}N_{r}}{R_{r}} \end{array}\right.$$

where *If* is the excitation current amplitude of the stator phase *q* winding.

To analyze the spatial order of the magnetokinetic potential, the Fourier series expansion of *B_q_* can be expressed as:

(2) $$B_{q}(\theta )=\overset{\infty }{\underset{v=1,3,5}{\sum }}B_{v}\sin\left(\frac{vD_{r}N_{s}}{\pi R_{r}Q}\right)\cos\left(\frac{vN_{s}\theta }{Q}\right)$$

where *Q* is the number of DDSRG phases, *N_s_* is the number of external stator poles, and the magnetodynamic potential coefficient *B_v_* is taken as:

(3) $$B_{v}=\frac{4N_{f}i_{q}}{n\pi }\;$$

**Figure 6:**
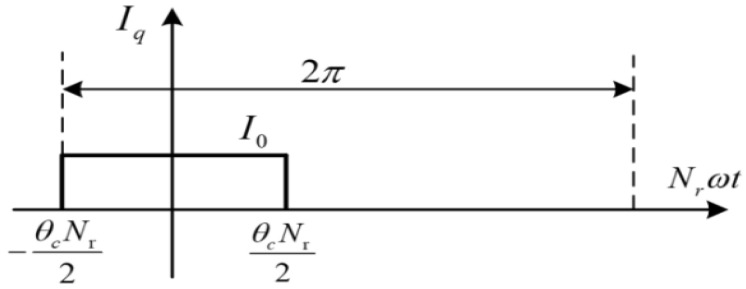
Time distribution of phase current (electrical angle).

Figure 6 shows the time distribution of the current in phase *q*. The expression of *I_q_* can be obtained from the distribution.

(4) $$I_{q}\left(\theta \right)=\left\{ \begin{array}{cc} I_{0} & -\frac{\theta_{c}}{2}N_{r}⩽N_{r}\omega t<\frac{\theta_{c}}{2}N_{r}\\ 0 & \frac{\theta_{c}}{2}N_{r}⩽N_{r}\omega t<2\pi -\frac{\theta_{c}}{2}N_{r} \end{array}\right.$$

where *θ*_c_ is the DDSRG conduction angle, and *ω* is the angular velocity of the motor. The Fourier expansion of the phase *q* current *i_q_* with time is:

(5) $$i_{q}(t)=\overset{\infty }{\underset{m=0}{\sum }}I_{m}\cos\left(vN_{r}\omega t\right)$$

where the value of *I_m_* is taken as:

(6) $$I_{m}=\left\{ \begin{array}{cc} \frac{I_{0}\theta_{c}N_{r}}{2\pi } & m=0\\ \frac{2I_{0}}{m\pi }\sin\frac{v\theta_{c}N_{r}}{2} & m=1,2,\cdot \cdot \cdot \end{array}\right.$$

Bringing Equations (3) and (5) into Equation (2), the Fourier series expansion of the magnetic potential *B_q_(θ*,*t)* of the phase *q* winding can be obtained by deducing:

(7) $$B_{q}(\theta ,t)=\overset{\infty }{\underset{v}{\sum }}\overset{\infty }{\underset{m}{\sum }}K_{vm}\cos(\frac{vN_{s}\theta }{2Q}\pm m\omega N_{r}t)$$

where *K_vm_* is the magnetic momentum coefficient associated with *v* and *m*. Therefore, the value of the magnetic momentum coefficient *K_vm_* is:

(8) $$K_{vm}=\left\{ \begin{array}{cc} \frac{N_{f}I_{0}\theta_{c}N_{r}}{v\pi^{2}}\sin\frac{vD_{r}N_{s}}{\pi R_{r}Q}\; & m=0\\ \frac{4N_{f}I_{0}}{mv\pi^{2}}\sin\frac{vD_{r}N_{s}}{\pi R_{r}Q}\sin\frac{m\theta_{c}N_{r}}{2}\; & m=1,2,3,\cdots \end{array}\right.$$

Bringing *n = vN_t_* into Equation (8), *K_nm_* can rewrite Equation (7) as follows [30]:

(9) $$B_{q}(\theta ,t)=\overset{\infty }{\underset{n}{\sum }}\overset{\infty }{\underset{m}{\sum }}K_{nm}\cos(n\theta \pm m\omega N_{r}t)$$

Therefore, the DDSRG magnetomotive force coefficient *K_nm_* varies with *n*,*m*. According to the operating principle of SRM, the different stator winding magnetomotive forces can be expressed as:

(10) $$\left\{ \begin{array}{l} B_{1}=\overset{\infty }{\underset{n}{\sum }}\overset{\infty }{\underset{m}{\sum }}K_{nm}\cos(n\theta \pm m\omega N_{r}t)\\ B_{2}=\overset{\infty }{\underset{n}{\sum }}\overset{\infty }{\underset{m}{\sum }}K_{nm}\cos\left[n\left(\theta +\frac{2\pi }{N_{s}}\right)\pm m\omega N_{r}\left(t-\frac{2\pi }{q\omega N_{r}}\right)\right]\\ B_{3}=\overset{\infty }{\underset{n}{\sum }}\overset{\infty }{\underset{m}{\sum }}K_{nm}\cos\left[n\left(\theta +\frac{4\pi }{N_{s}}\right)\pm m\omega N_{r}\left(t-\frac{4\pi }{q\omega N_{r}}\right)\right]\\ \;\;\cdot \cdot \cdot \end{array}\right.$$

Because of the special characteristics of the convex pole structure of the SRM stator and rotor, the saturation characteristics of the motor magnetic circuit need to be considered. At higher currents, the linearity of the magnetic permeability decreases as the rotor rotation angle becomes larger. Due to the phenomenon of magnetic circuit saturation, the magnetic chain appears nonlinear when the current grows, so an accurate solution of the motor permeability is very important. Since the DDSRG stator is not slotted, and the air-gap distance between the stator teeth is large, the permeability between the stator teeth can be neglected. Therefore, the Fourier expansion of the air-gap permeability function Λ_r_*(θ*,*t)* is:

(11) $$\Lambda_{r}(\theta ,t)=\Lambda_{r0}+\overset{\infty }{\underset{z=1}{\sum }}\Lambda_{rz}\cos\left(z\omega t-\frac{zN_{s}\theta }{2Q}\right)$$

(12) $$\Lambda_{r0}=\frac{1}{\pi }\frac{\mu_{0}}{g_{o}}\left(\pi +\frac{z\pi N_{r}}{4}\right)$$

where Λ_rz_ is the coefficient of magnetic permeability as:

(13) $$\begin{array}{l} \Lambda_{rz}=\frac{1}{\pi }\int_{-\pi }^{\pi }\Lambda (\theta )\cos(z\theta )d\theta \\ \;\;=\frac{2}{z\pi }\frac{\mu_{0}}{g_{o}}\left[\sin\left(\frac{zD_{r}N_{r}}{R_{r}}\right)-\sin\left(\frac{z\pi N_{r}}{4}-\frac{zD_{r}N_{r}}{R_{r}}\right)\right] \end{array}$$

where *μ*_0_ is the vacuum permeability, and *g_o_* is the outer fixed-rotor radial air-gap distance. Therefore, it can be found that the rotor permeability Λr*(θ*,*t)* is related to the rotor position angle *θ*, and the rotor pole arc coefficient will have an effect on the permeability.

According to the electromagnetic field principle, the radial flux density of phase *q* winding is *B_rq_(θ*,*t)*:

(14) $$B_{rq}(\theta ,t)=B_{q}(\theta ,t)\times \Lambda_{r}(\theta ,t)$$

Combining the Maxwell stress equation, it can be seen that the combined force and momentum within a given volume and magnetic mass are equivalent to the sum force of the stator surface tension. By further analysis, it can be obtained that the tangential flux density is much smaller than the radial flux density, so the radial electromagnetic force *F*_r_ can be expressed as:

(15) $$F_{r}=\frac{B_{\mathrm{rq}}^{2}-B_{\mathrm{tq}}^{2}}{2\mu_{0}}\approx \frac{B_{\mathrm{rq}}^{2}}{2\mu_{0}}$$

where *B_tq_* is the tangential flux density; the analytical formula of *F*_r_*(θ,t)* can be obtained by combining Equations (11) and (12).

(16) $$F_{r}(\theta ,t)=\frac{{\left(B_{q}(\theta ,t)\times \Lambda_{r}(\theta ,t)\right)}^{2}}{2\mu_{0}}=\left[\begin{array}{l} B_{q}^{2}(\theta ,t)+\Lambda_{r}^{2}(\theta ,t)\\ +2\times B_{q}(\theta ,t)\times \Lambda_{r}(\theta ,t) \end{array}\right]$$

Based on the principle of minimum reluctance, the tangential magnetic density decreases continuously and the radial magnetic density increases continuously during the process of turning from the maximum reluctance position to the minimum reluctance position under the influence of electromagnetic force. Combining with Equation (13), it can be seen that the ERF is increasing with the rotor position during the rotation from the maximum reluctance position to the minimum reluctance position. In this paper, the flux density and the ERF of the inner stator circuit of DDSRG are studied in the same way as that of the outer stator rotor, and the calculation process of the outer stator rotor can be referenced.

## 4. DDSRG Electromagnetic and Vibration Characterization

### 4.1. Electromagnetic Characteristic Calculation and Analysis

**Figure 7:**
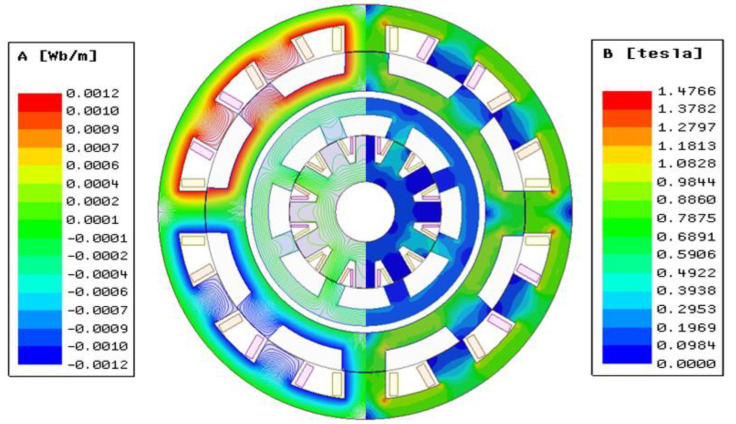
DDSRG magnetic induction line distribution and magnetic density cloud diagram.

**Figure 8:**
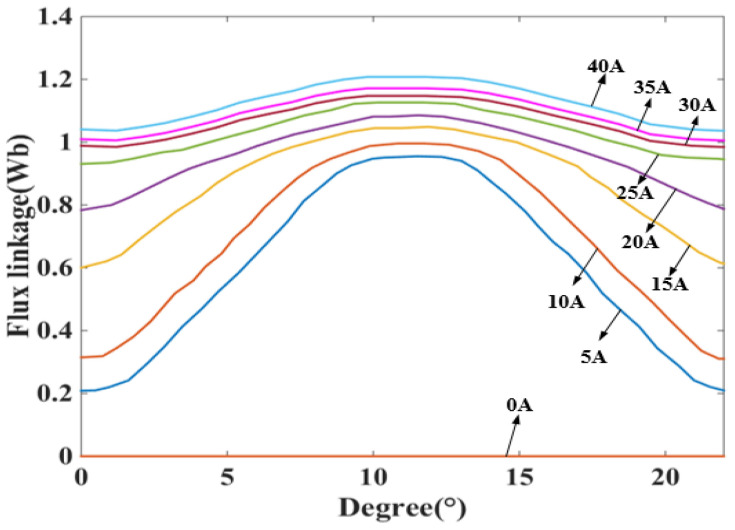
Variation of magnetic chain at different angles.

Due to the dual-stator structure of the DDSRG, analysis of the air-gap magnetic density needs to be performed simultaneously for both the inner and outer air gaps. In this paper, we use the finite element software Maxwell for magnetic field analysis and establish the finite element model of DDSRG with 12/8/8/12 poles, and the stator and rotor cores are made of non-oriented silicon steel sheet material DW540-50, and the rated voltage of the external circuit is 220 V, and the rated voltage of the internal circuit is 100 V. Figure 7 shows the magnetic inductance line distribution and magnetic density cloud of DDSRG through the magnetic inductance line distribution It can be seen that during the process of energizing one of the phases, the internal and external stator will excite the four tooth poles, respectively. According to the magnetic density cloud diagram, it can be observed that there is a large flux density in the stator teeth and stator yoke where the excitation is generated, and there is no magnetic leakage or saturation phenomenon. The magnetic flux in the inner and outer circuits is evenly distributed, so the magnetic separation plate plays a corresponding role in preventing electromagnetic interference when the inner and outer circuits are working alternately, which ensures the normal operation of the power-generation system. Due to the characteristics of the SRG stator–rotor biconvex pole structure, the stator–rotor overlap process is prone to magnetic saturation problems. The accuracy of the generator’s electromagnetic force characteristics is ensured by performing finite element calculations on the magnetic saturation characteristics of the 2D DDSRG model prior to electromagnetic force calculations. Figure 8 shows the variation curves of the single-tooth pole magnetic chain of the DDSRG at different currents at rated speed, and it can be observed that the magnetic chain varies non-linearly into saturation as the current amplitude increases. By combining the results of the finite element settlement of the magnetic chain, the simulation of the electromagnetic force is provided with basic support.

**Figure 9:**
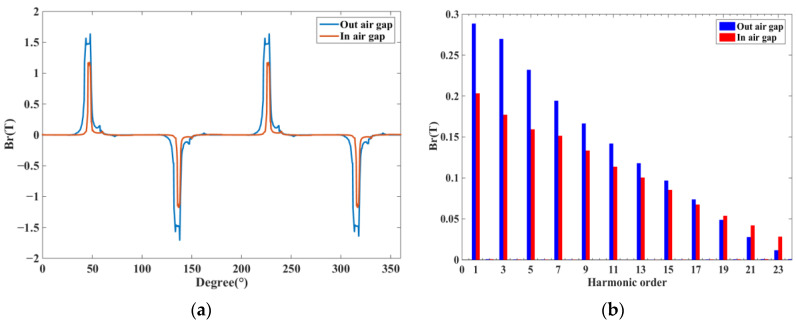
Radial magnetic density of the air-gap space inside the outer air gap of DDSRG. (**a**) Magnetic density waveform diagram. (**b**) Harmonic decomposition.

The radial component of the magnetic flux density plays an important role in the ERF, and the ERF of the DDSRG stator teeth can be obtained by integrating the radial component of the air-gap magnetic density over the stator teeth surface. Figure 9 shows the air-gap magnetic density waveform and harmonic analysis of the electromagnetic system inside and outside the DDSRG at rated speed. Since each phase winding has four stator teeth, the magnetic density waveform plot in Figure 9a has four peaks, alternating positive and negative. The radial air-gap magnetic density amplitude of the outer air gap is larger than that of the inner air gap because of the higher excitation voltage of the external circuit, but the waveforms are similar due to the same operating principle. The spatial harmonic decomposition of Figure 9b can be derived from the amplitude of the radial air-gap magnetic density mainly considering the lower order harmonics.

**Figure 10:**
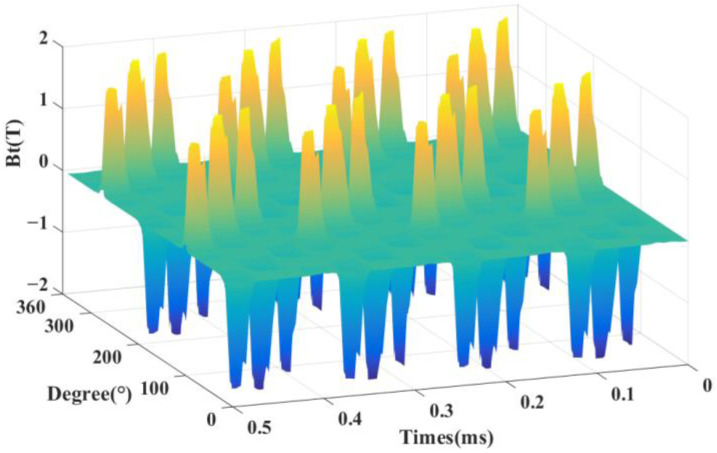
Radial magnetic density–rotor position–time waveform of external air gap of DDSRG.

**Figure 11:**
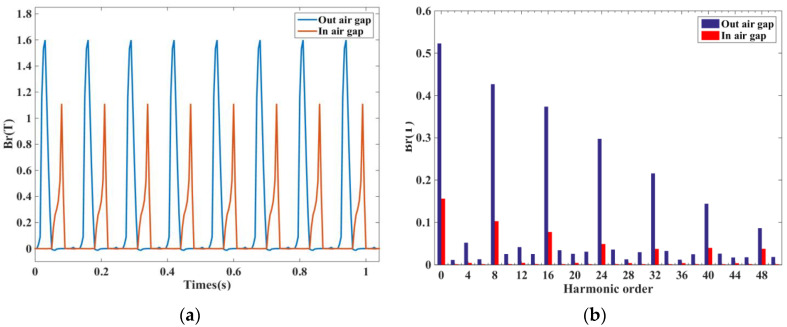
Radial magnetic density of air-gap time inside and outside the DDSRG. (**a**) Magnetic density. (**b**) Harmonic decomposition.

In order to see the radial variation of the DDSRG air-gap magnetic density more clearly, taking the outer air-gap as an example, Figure 10 shows the DDSRG outer air-gap radial magnetic density–rotor position–time waveform. It can be observed along the spatial axis that when a phase is excited, four corresponding radial component peaks appear. Observed along the time axis, 12 peaks appear in one excitation cycle, indicating alternating operation of the three phases, which is in accordance with the generator stator structure characteristics. As shown in Figure 11 for the air-gap time characteristics of the DDSRG and the time domain decomposition spectrum obtained by FFT, the poles appear mainly at a time order of 8*N* (*N* = 0, 1, 2…)Hz.

**Figure 12:**
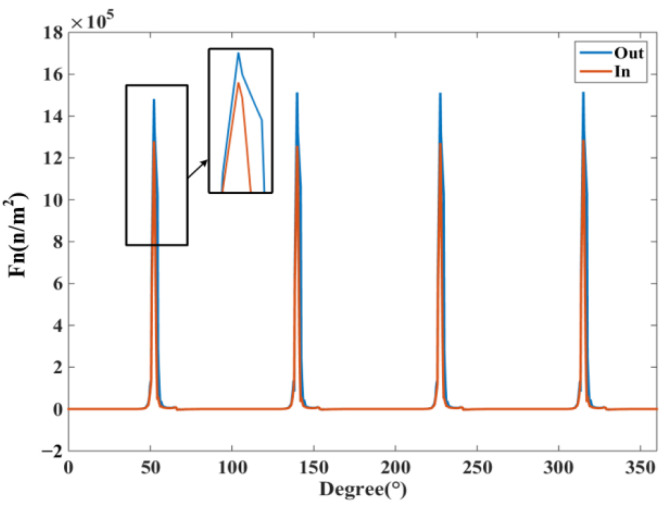
ERF waveform with rotor position.

**Figure 13:**
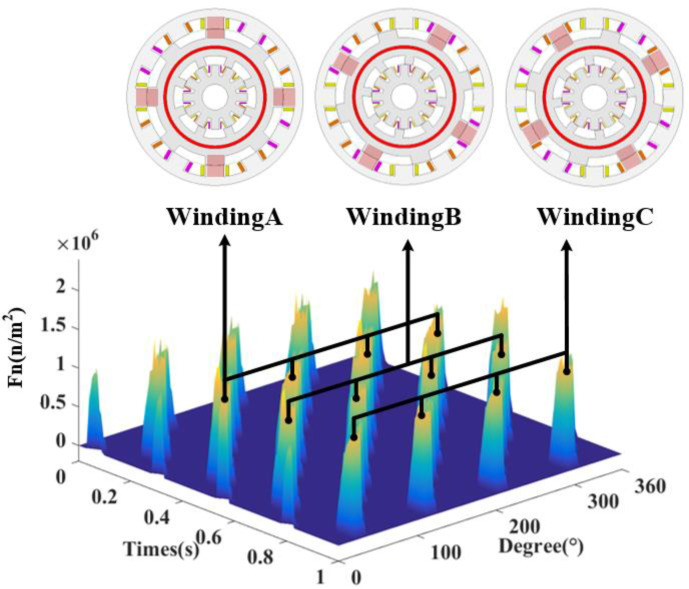
Radial electromagnetic force–rotor position–time waveform of DDSRG.

Figure 12 shows the ERF waveform, and compared with the spatial distribution of radial magnetic density, it can be found that the ERF has a larger amplitude at the larger radial magnetic density, which is consistent with the theoretical derivation. According to the enlarged plot, it can be found that the ERF distribution is not uniform mainly in the front of the stator teeth, while the amplitude at the back is small. In order to observe the ERF time and space distribution more easily, Figure 13 shows the DDSRG radial electromagnetic force–rotor position–time waveform, and it can be observed along the time axis that the ERF is alternately distributed with the rotor position change during an operating cycle. When each phase winding is operated, there are four peaks corresponding to it.

**Figure 14:**
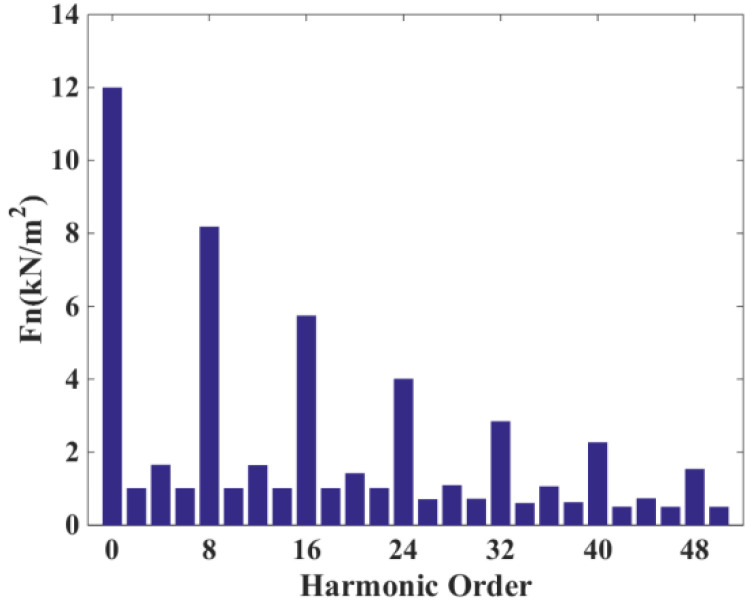
Radial electromagnetic force time decomposition of the DDSRG.

Figure 14 shows the time decomposition of the ERF of the DDSRG with a fundamental frequency *f*_0_ of 160 Hz at rated speed, and the fundamental frequency *f*_0_ is calculated as follows

(17) $$f_{0}=\frac{n\cdot N_{r}\cdot \;\left(N_{s}/2\right)}{60}$$

where *n* is the rotational speed, and N_s_ and N_r_ are the number of stator poles and rotor poles of the generator. Since the ERF acting on the rotor is a combined force, N_s_/2 needs to be added as a factor to the switching frequency of each phase. Usually, during the electromagnetic vibration analysis of a motor, strong vibration and noise are generated when the structure’s intrinsic frequency and the electromagnetic force frequency are the same or similar. To avoid its resonance situation, the amplitude and frequency of ERF must be obtained. By making FFT analysis of the distribution of the radial electromagnetic force as a function of time, the extreme moments are obtained as 8 *f_0_N*(*N* = 0, 1, 2…)Hz, etc.

**Figure 15:**
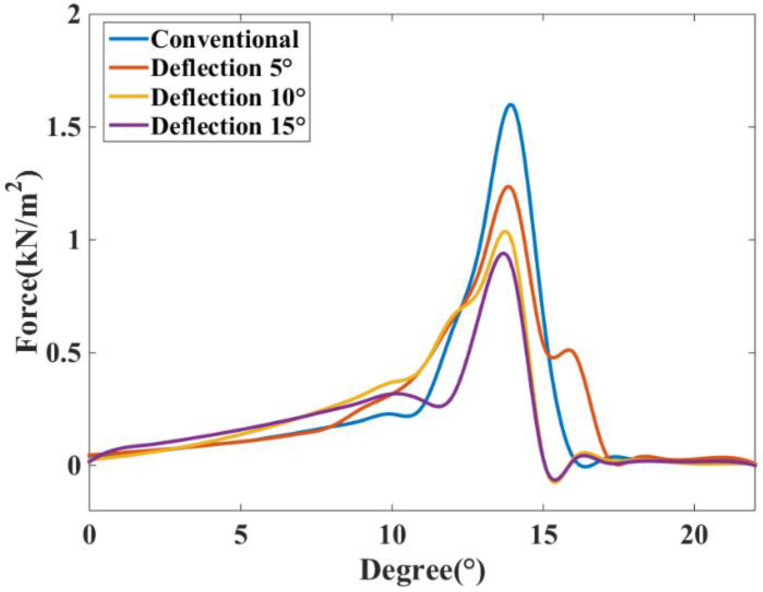
Radial force distribution under different deflection angles.

As the DDSRG undergoes deflection operation, the overall power-generation process of the generator changes. The ERF of the stator teeth is simulated at different angles of deflection, and Figure 15 shows the radial force distribution at different deflection angles. As the deflection angle increases, the peak value of the ERF appears to decrease, and the overall trend is down due to the reduction of stator–rotor overlap area after deflecting a certain angle. The peak of the ERF decreases and the overall waveform distribution is similar to that of normal operation, but the generator efficiency also decreases.

### 4.2. Modal and Vibration Analysis of DDSRG

The electromagnetic vibration is not only related to the electromagnetic force between the air gap but also related to the inherent frequency of the stator. When the frequency of the electromagnetic force wave of a certain order is the same as or close to the inherent vibration frequency of the stator, resonance will occur, resulting in large electromagnetic noise. Since the numerical accuracy of the analytical method is poor, and the accuracy of the finite element method is high, this paper uses the finite element method to calculate. The finite element method is based on the Lagrange’s equation for the calculation of the stator inherent frequency, and the generalized coordinate system is used to calculate the coordinate system. Usually, in the finite element calculation, the unit potential and kinetic energy are:

(18) $$T=\frac{1}{2}{\dot{\delta }}^{T}\int_{ve}N^{T}\rho_{e}Ndv\dot{\delta }$$

(19) $$U=\frac{1}{2}{\dot{\delta }}^{T}\int_{ve}B^{T}DBdv\delta$$

where *δ* is the displacement vector of the unit node; *ρ_e_* is the mass density; *T* is the transpose sign; *N* is the form function; *B*, *D* is the strain matrix and elasticity matrix. In turn, the unit mass matrix and the unit stiffness matrix are obtained as follows:

(20) $$M_{e}=\int_{ve}N^{T}\rho_{e}Ndv$$

(21) $$K_{e}=\int_{ve}B^{T}DBdv$$

According to Hamilton’s basic principle and the relationship between strain–displacement and stress–strain, the equation of motion of the unit after the motor is discretized is obtained as follows.

(22) $${\left[M\right]}_{e}{\left\{\ddot{u}\right\}}_{e}+{\left[R\right]}_{e}{\left\{\dot{u}\right\}}_{e}+{\left[K\right]}_{e}{\left\{u\right\}}_{e}={\left\{F\right\}}_{e}$$

where [*R*] is the damping matrix, [*M*] is the mass matrix, [*u*] is the displacement vector, [*K*] is the stiffness matrix, and [*F*] is the force vector. The finite element calculation can be used to obtain the intrinsic frequency of the motor as well as the vibration mode, but the analysis yields the intrinsic frequency of the motor, which is usually undamped, free vibration. Therefore, in Equation (22), make {*F*} = {0} and replace the time derivative with *jω*. The corresponding eigenvalues of the undamped free vibration mode of the motor are calculated as:

(23) $$\left(\left[K]-\omega^{2}[M\right]\right)\{u\}=\{0\}$$

Based on the linear theory, it is known that the sufficient necessary condition for the existence of non-zero solutions is that the coefficient of {*u*} is 0. According to the solution of the vector corresponding to the non-zero solutions, the intrinsic frequency and intrinsic vibration pattern of the motor can be obtained.

According to the mechanical structure of the DDSRG, the internal and external stator models are established, and modal analysis is performed. Since the stator core is formed by laminating silicon steel sheets, the material used to perform the analysis is different from that of the solid core, and its influence on the material properties should be considered comprehensively. The stator core is analyzed as accurately as possible, so the density of the stator core is the product of the actual material density and the lamination factor. The actual material properties of the stator core are shown in Table 2.

**Figure 16:**
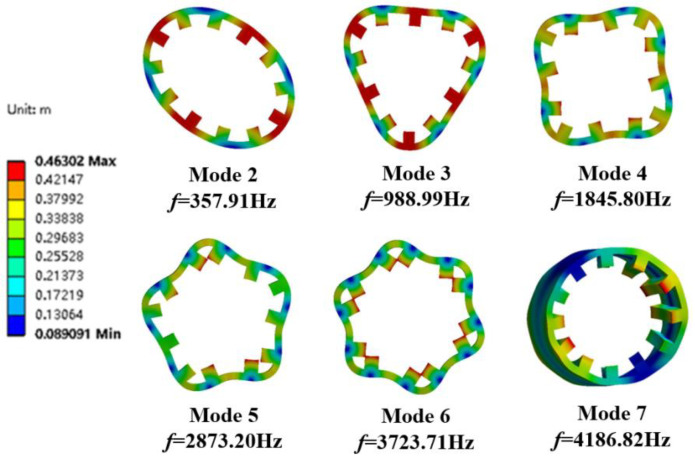
External stator modal analysis cloud diagram.

**Figure 17:**
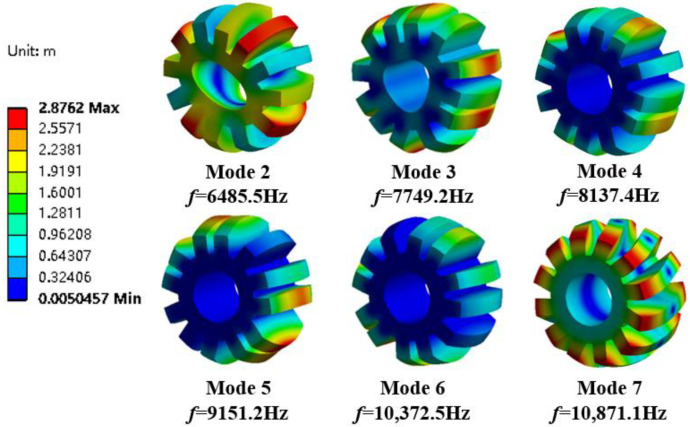
Internal stator modal analysis cloud diagram.

The modal analysis of the stator housing of the motor is performed without setting constraints and boundary conditions during the simulation analysis. The radial and axial dimensions of the vibration model have a large influence on the results because the stator is made of a stack of silicon steel sheets with an uneven overall distribution and strong material orthogonality. The parameters of the internal and external stator simulation model are shown in Figure 1, with a stator axial length of 80 mm. the rotor stiffness is much stronger than the stator strength when electromagnetic force waves act on the 3D finite element simulation model, so the focus is on the generator stator structure vibration. The 3D finite element models of the external and internal stator of the DDSRG are created by the finite element software, and the simulation conditions are simulated within 15 kHz. Due to the special structure of the stator spherical surface, the modal structure is more complex but varies regularly. According to the modal analysis, the inherent frequencies of each order of the inner and outer stator and the corresponding vibration clouds are shown in Figure 16 and Figure 17. From the vibration cloud diagram, it can be observed that the stator teeth are subjected to large forces, while the stator yoke and stator slot are subjected to relatively small forces. Therefore, the main vibrational deformation occurs mostly in the stator teeth, and the deformation is mainly elliptical, triangular, quadrangular, pentagonal, etc. From the stator axial direction, to observe the two ends of the deformation is larger, and due to the outer stator teeth for the spherical structure, the length of the two ends of the teeth is greater than the length of the central stator teeth. On the contrary, the length of the middle part of the stator tooth of the inner stator is longer than the two ends, so the larger part of the inner stator deformation is mainly concentrated in the middle of the stator tooth.

**Figure 18:**
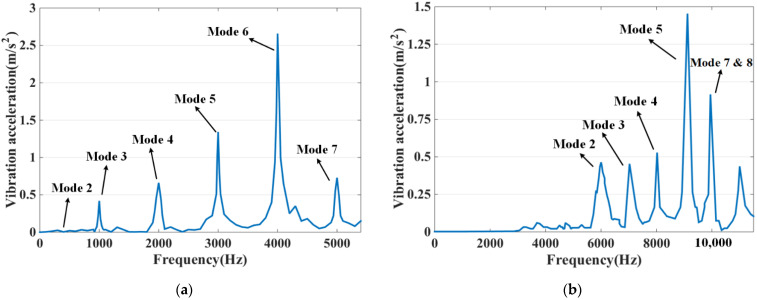
Vibrating surface acceleration at rated speed. (**a**) Outer stator. (**b**) Inner stator.

**Figure 19:**
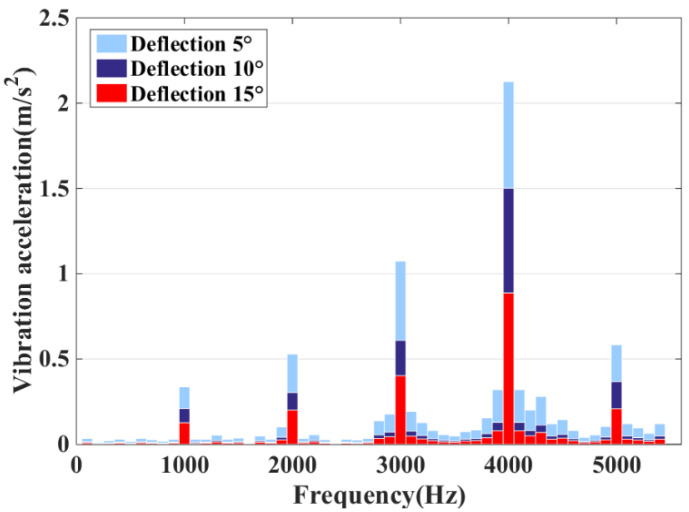
Vibrating surface acceleration at different deflection angles.

The modal analysis has been carried out above on the basis of which the modal vibration pattern is superimposed to analyze its vibration characteristics. The harmonic response analysis allows a comprehensive and effective analysis of the intrinsic modal and ERF of the DDSRG. By combining the electromagnetic–structural coupling analysis process in Figure 1, the calculated ERF on the inner surface of the stator is used as the excitation source and coupled to the stator teeth for harmonic response analysis in the frequency domain. The ERF can be decomposed into a series of harmonics, and resonance occurs when a certain order of harmonics is similar to or the same as the intrinsic frequency of the motor. Figure 18 shows the vibration acceleration distribution of the outer and inner stator surfaces at rated speed without deflection, and the resonance is caused when the frequency of the corresponding harmonic is close to the intrinsic frequency of the stator. When the DDSRG is operated in deflection, the ERF decreases with the increase of deflection angle. Figure 19 shows the surface acceleration of stator vibration at different deflection angles. With the increase of deflection angle, the amplitude of the ERF vibration response decreases significantly, but the vibration frequency does not change significantly.

## 5. Experimental Verification

**Figure 20:**
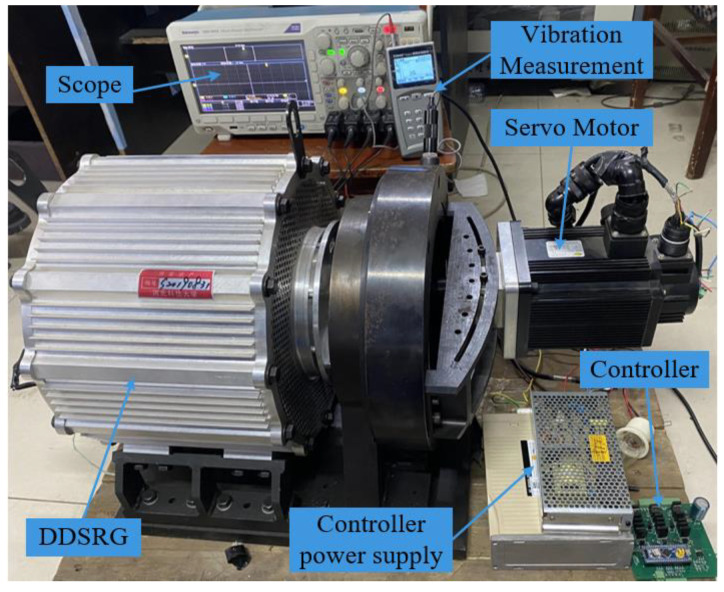
DDSRG experimental platform.

**Figure 21:**
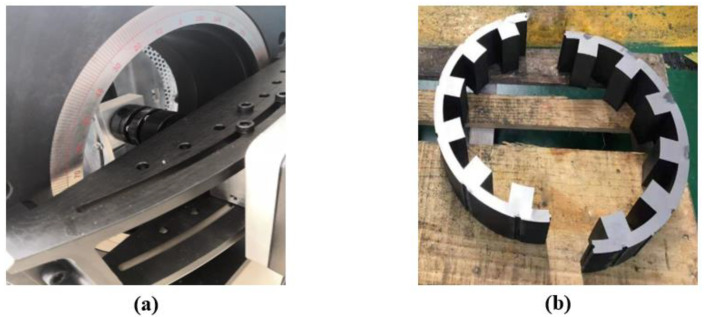
Parts diagram of DDSRG. (**a**) Generator deflection scale disc. (**b**) Stator structure.

After the above analysis, as shown in Figure 20, the DDSRG experimental platform is established to verify the generator operating characteristics and vibration law. The experimental platform includes a DDSRG test prototype, a controller, a servo motor, a switching power supply, an oscilloscope, and a vibration meter. A servo motor is used to drive the DDSRG to generate electricity, and a light bulb is used to replace the load and determine the power-generation status by the brightness. A DC power supply is used to provide excitation for the generator, and the power is generated by another excitation method to ensure the stability of the power-generation system. Real-time data on the electromagnetic characteristics of the DDSRG can be measured by an oscilloscope during the power-generation process. The vibration response of the motor at different positions is measured and collected using the probe of the vibrometer TIME7231, and the measurement results are stored by spectrum and curve. Figure 21a shows the deflection dial of the DDSRG, with the output shaft supported by a fixed guide for deflection motion, and the dial can visually show the deflection angle. Figure 21b shows the rotor structure of the DDSRG. The rotor material uses a DW465-50 silicon steel sheet with a lamination factor of 0.95, and the stator tooth surface has a special spherical structure to provide support for multi-degree-of-freedom operation.

**Figure 22:**
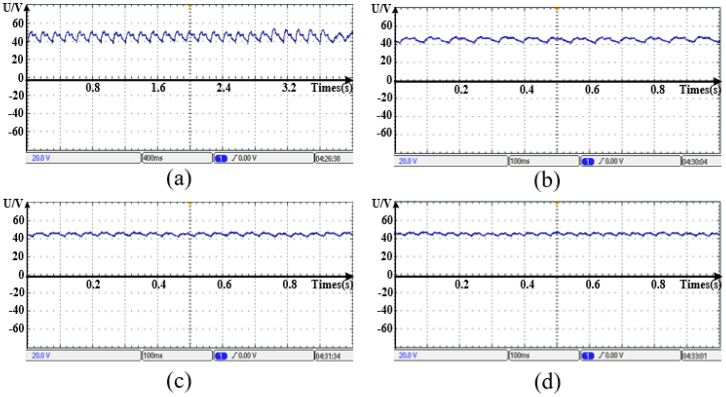
Voltage waveforms of DDSRG at different speeds. (**a**) *n* = 50 rpm. (**b**) *n* = 100 rpm. (**c**) *n* = 150 rpm. (**d**) *n* = 200 rpm.

**Figure 23:**
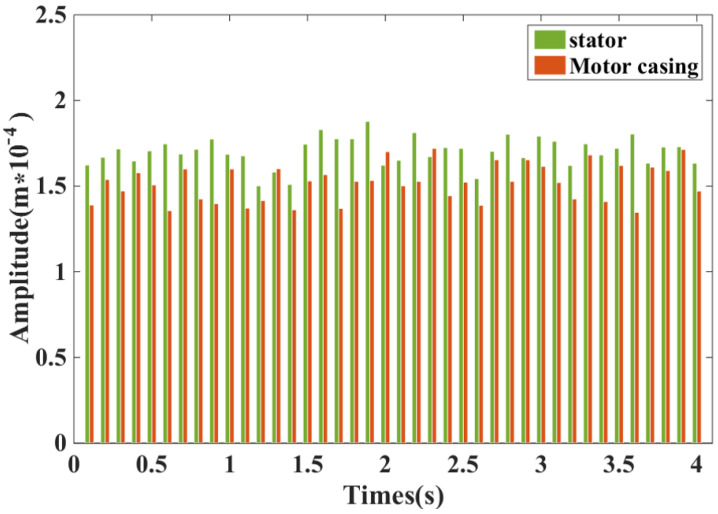
Casing and stator vibration characteristics of DDSRG at rated speed.

Figure 22 shows the voltage waveforms of the DDSRG at different rotational speeds. By observation, it is obtained that the voltage fluctuation is larger at lower rotational speeds and gradually decreases as the rotational speed gradually increases and gradually smooths out at rated speed. Because of the convex structure of the generator rotor teeth, the flux change in the generator electromagnetic circuit is amplified at low speed, but when the rated speed is increased, the voltage amplitude and fluctuation are gradually stabilized, which verifies the stability and feasibility of the DDSRG operation. The vibration characteristics of the stator and motor casing are observed in Figure 23 by measuring the stator and motor casing at rated speed with a vibrometer, and it is found that the casing has a restraining effect on the stator vibration.

## 6. Conclusions

In this paper, the time and frequency domains of unbalanced electromagnetic forces of a DDSRG with a rated power of 2.5 kW are analytically calculated, and the vibration characteristics of the generator are simulated and analyzed for both self-rotating operation and deflecting operation and finally verified and compared by finite element analysis and experimental results. DDSRG allows for a multi-degree of freedom operation, and the excitation current action produces particularly significant ERF pulsations during generator operation. The calculation of the electromagnetic field of the DDSRG at different deflection angles is carried out using finite element software, and the distribution law of the ERF in time and space is analyzed to verify that the main amplitude order of the generator ERF acting on the stator is 8 *f_0_*N(*N* = 0, 1, 2…). The modal analysis of different stator structures of switched reluctance motors with corresponding materials shows that the vibration order is more intense in the order 2–7. By combining the coupling analysis of electromagnetic-vibration, the vibration response of DDSRG was calculated at the benchmark of rated speed of 200 rpm. The results of harmonic response spectrum analysis corresponded to the modal analysis, and the electromagnetic force wave near the intrinsic frequency had a greater influence on the generator vibration. Finally, by establishing the DDSRG vibration measurement experimental platform, the power-generation characteristics and vibration response were tested, and the experimental results were basically consistent with the finite element analysis results.

## Figures and Tables

**Table 2:** Motor material parameters.

Parameter	Value
Modulus of elasticity	2.06 × 10^11^ Pa
Poisson’s ratio	0.3
Density	7884 kg/m^3^

## Data Availability

Not applicable.
